# SOX30, a target gene of miR-653-5p, represses the proliferation and invasion of prostate cancer cells through inhibition of Wnt/β-catenin signaling

**DOI:** 10.1186/s11658-019-0195-4

**Published:** 2019-12-23

**Authors:** Qiang Fu, Zhenye Sun, Fan Yang, Tianci Mao, Yanyao Gao, He Wang

**Affiliations:** Department of Urology, Tangdu Hospital, The Fourth Military Medical University, 1 Xinsi Road, Xi’an, 710038 Shaanxi China

**Keywords:** SOX30, MiR-653-5p, Prostate cancer, Wnt/β-catenin

## Abstract

**Background:**

Sex-determining region Y-box containing gene 30 (SOX30) is a newly identified tumor-associated gene in several types of cancer. However, whether SOX30 is involved in the development and progression of prostate cancer remains unknown. This study investigated the potential role of SOX30 in prostate cancer.

**Methods:**

Prostate cancer cell lines and a normal prostate epithelial cell line were used for the experiments. The expression of SOX30 was determined using quantitative real-time PCR and western blot analysis. The malignant cellular behaviors of prostate cancer were assessed using the Cell Counting Kit-8, colony formation and Matrigel invasion assays. The miRNA–mRNA interaction was validated using the dual-luciferase reporter assay.

**Results:**

SOX30 expression was lower in cells of prostate cancer lines than in cells of the normal prostate epithelial line. Its overexpression repressed the proliferation and invasion of prostate cancer cells. SOX30 was identified as a target gene of microRNA-653-5p (miR-653-5p), which is upregulated in prostate cancer tissues. MiR-653-5p overexpression decreased SOX30 expression, while its inhibition increased SOX30 expression in prostate cancer cells. MiR-653-5p inhibition also markedly restricted prostate cancer cell proliferation and invasion. SOX30 overexpression or miR-653-5p inhibition significantly reduced β-catenin expression and downregulated the activation of Wnt/β-catenin signaling. SOX30 knockdown significantly reversed the miR-653-5p inhibition-mediated inhibitory effect on the proliferation, invasion and Wnt/β-catenin signaling in prostate cancer cells.

**Conclusions:**

These results reveal a tumor suppressive function for SOX30 in prostate cancer and confirmed the gene as a target of miR-653-5p. SOX30 upregulation due to miR-653-5p inhibition restricted the proliferation and invasion of prostate cancer cells, and this was associated with Wnt/β-catenin signaling suppression. These findings highlight the importance of the miR-653-5p–SOX30–Wnt/β-catenin signaling axis in prostate cancer progression.

## Background

Prostate cancer is a common malignant tumor of the urinary system in the male population worldwide [1]. According to Cancer Statistics, 2019 [1], prostate cancer accounts for 20% of all new cancer diagnoses in males (the highest incidence rate). Despite advances in its detection and treatment, it remains the second leading cause of cancer-related deaths [1, 2].

Radical prostatectomy and/or radiation are the standard primary treatments for patients with localized prostate cancer, while androgen suppression is the main therapy for recurrent disease and/or advanced prostate cancer [3]. Although androgen suppression therapy is initially effective, almost all prostate cancer patients ultimately progress to metastatic castration-resistant prostate cancer [4]. The median overall survival for metastatic castration-resistant prostate cancer patients ranges from 13 to 32 months with a 5-year survival rate less than 15% [5].

Prostate cancer molecular pathogenesis is very complex, involving multiple genetic alterations [6]. However, despite extensive investigations, we remain far from a full understanding of the mechanism. Further investigations of the molecular underpinnings of prostate cancer’s occurrence and progression will help to identify new targets for the development of effective and promising prostate cancer treatments.

Sex-determining region Y-box (SOX) proteins, a family of transcription factors that contain domains consisting of high mobility groups, play a pivotal role in a wide range of biological processes [7–9]. Notably, SOX family members are critical regulators in the development and progression of various cancers, functioning as either oncogenes or tumor suppressors [10].

SOX30 is a newly identified cancer-related SOX member that exerts a significant impact on multiple cancer types [11, 12]. Low SOX30 expression occurs in lung cancer, hepatocellular carcinoma, acute myeloid leukemia, ovarian cancer and bladder cancer [12–17]. Thus, it has potential biomarker as a for diagnosis and prognosis. Moreover, SOX30 inhibits tumor cell proliferation and invasion, and promotes tumor cell apoptosis, suggesting a tumor-suppressive role [18, 19]. Therefore, it could have promise as an anticancer target.

MicroRNAs (miRNAs) are a subtype of noncoding RNAs that are composed of 19–25 nucleotides generated from a series of cleavage processes [20]. They play an important role in regulating the expression of protein-coding genes, mostly through binding to the 3′-untranslated region (3′-UTR) of target messenger RNA (mRNA) [20, 21]. MiRNA binding to mRNA can result in mRNA degradation and translational inhibition, which is how these molecules inhibit gene expression. MiRNAs probably regulate various biological functions by negatively regulating gene expression. They also participate in cancer development and progression [22, 23]. Several lines of evidence indicate that various miRNAs are dysregulated in prostate cancer, contributing to its tumorigenesis, and that they could serve as potential diagnostic and prognostic biomarkers as well as promising therapeutic anticancer targets [24–26]. MiRNA-regulated gene networks are an exciting area of research for prostate cancer therapies.

To date, little is known about the role of SOX30 in prostate cancer. This study investigated its expression, biological function and regulatory mechanism in this malignancy. We found that SOX30 levels were significantly lower in prostate cancer cells than in normal prostate epithelial cells. SOX30 overexpression in prostate cancer cell lines markedly reduced their proliferative ability and invasive potential.

Interestingly, SOX30 was identified as a miR-653-5p target gene. MiR-653-5p expression is elevated in prostate cancer cells and its inhibition significantly restricts the proliferation and invasion of these cells. Here, the inhibitory effect of SOX30 overexpression or miR-653-5p inhibition on prostate cancer cell proliferation and invasion was associated with a suppressive effect on the activation of Wnt/β-catenin signaling. Our results reveal a tumor-suppressive function for SOX30 in prostate cancer and highlight the importance of the miR-653-5p–SOX30–Wnt/β-catenin signaling axis in prostate cancer progression.

## Materials and methods

### Cell culture

Human prostate cancer cell lines PPC-1, PC-3, LNCaP and DU-145, and a normal prostate epithelial cell line (RWPE-1) were purchased from the American Type Culture Collection (ATCC). Culture was performed according to the manufacturer’s recommended methods. Briefly, PPC-1 and LNCaP cells were grown in RPMI 1640 (Gibco) that contained 10% fetal bovine serum (FBS). PC-3 cells were grown in Ham’s F-12 K Medium (Gibco) supplemented with 10% FBS. DU-145 cells were cultured in minimum essential medium (Gibco) that contained 10% FBS. RWPE-1 cells were maintained in keratinocyte serum-free medium (Gibco). 293 T cells were donated by the Cell Bank of the Chinese Academy of Sciences and cultured in Dulbecco’s modified Eagle medium (DMEM; Gibco) supplemented with 10% FBS. All cells were maintained at 37 °C in a humidified incubator with 5% CO_2_.

### Cell transfection

The complementary DNA (cDNA) sequences of the SOX30 open reading frame were subcloned into the pcDNA3.1 plasmid to generate SOX30 expression plasmids. The oligonucleotides of the miR-653-5p mimics, inhibitor and negative control (NC), and the SOX30 silencing RNA (siRNA) were purchased from RiboBio. The plasmids and oligonucleotides were transfected into cells using Lipofectamine 3000 (Invitrogen).

### Quantitative real-time PCR analysis

Total RNA was isolated using TRIzol Reagent (Invitrogen) and reverse transcribed into cDNA using the PrimeScript RT Reagent Kit (Takara) according to the manufacturer’s protocols. SOX30 transcription levels were determined using PowerUp SYBR Green Master Mix (Applied Biosystems), with glyceraldehyde 3-phosphate dehydrogenase (GAPDH) as the internal control. Small RNA-containing total RNA was extracted and purified with the mirVana miRNA Isolation Kit (Ambion) and converted into cDNA using the Taqman miRNA Reverse Transcription Kit (Applied Biosystems) following the manufacturer’s instructions. MiR-653-5p expression was determined with TaqMan Fast Advanced Master Mix (Applied Biosystems), using U6 as the internal control. Target gene expression was determined with the 2^−ΔΔCt^ method.

### Western blot analysis

Cells were lysed in lysis buffer (Beyotime Biotechnology) that contained a phosphatase inhibitor cocktail. The supernatant was collected by centrifugation, and the protein concentration was determined with the BCA Protein Assay Kit (Beyotime Biotechnology). Equal amounts of total protein were loaded onto sodium dodecyl sulfate polyacrylamide (SDS-PAGE) gels and resolved by electrophoresis. The separated proteins were transferred to a polyvinylidene fluoride membrane followed by incubation with 5% non-fat milk solution for 1 h at room temperature. Next, the membrane was probed with primary antibodies against the target proteins at 4 °C overnight. The antibodies were rabbit polyclonal anti-SOX30 (Invitrogen; #PA5–40508), rabbit monoclonal anti-GAPDH (Abcam; #ab9485), and rabbit monoclonal anti-active β-catenin (Cell Signaling Technology; #19807). Subsequently, the membrane was incubated with horseradish peroxidase-conjugated goat anti-rabbit IgG (Abcam; #ab205718) for 1 h at room temperature. Finally, an enhanced chemiluminescence kit (Millipore) was used to visualize the protein bands. Bands of interest were quantified using Image-Pro Plus 6.0 software.

### Cell proliferation assay

Cell proliferation was determined with the Cell Counting Kit-8 (CCK-8) assay. In brief, prostate cancer cells were seeded into a 96-well plate and transfected with SOX30 expression vector, miR-653-5p mimics or inhibitor. After 48 h, the cells were treated with 10 μl/well CCK-8 solution (Beyotime Biotechnology). Cells were then cultured for 2 h at 37 °C before measurement of absorbance at 490 nm with a microplate reader (BioTek Instruments).

### Colony formation assay

Cells were transfected with SOX30 expression vector for 48 h and then resuspended in medium that contained 0.3% agarose. The transfected cells were then seeded into six-well plates pre-coated with growth medium that contained 0.6% agarose. Cells were cultured for 14 days at 37 °C. For colony visualization, cells were fixed with 4% paraformaldehyde and stained with 0.1% crystal violet. After washing with phosphate-buffered saline (PBS), the colonies were observed under an optical microscope.

### Matrigel invasion assay

Transfected cells were suspended in 200 μl serum-free medium and plated into the upper chambers of Matrigel Invasion Chambers (BD Biosciences). The lower chambers were filled with 500 μl normal medium that contained 10% FBS. Cells were cultured for 24 h at 37 °C. The residual cells on the upper surface of the membrane were wiped away, and the invasive cells on the lower side of the membrane were fixed with 4% paraformaldehyde. For visualization, the cells were stained with 0.1% crystal violet, and the invasive cell number was counted with an optical microscope.

### Dual-luciferase reporter assay

To detect whether SOX30 is an miR-653-5p target gene, a SOX30 3′-UTR with the normal miR-653-5p-binding site or a mutant binding site was inserted into the pmirGLO reporter vector (Promega). The constructed reporter vectors were co-transfected with miR-653-5p mimics into 293 T cells and incubated for 48 h. Subsequently, cells were harvested and lysed, and the luciferase activity was measured with a Dual-Luciferase Reporter System (Promega) according to the manufacturer’s manual.

### Statistical analysis

All data are expressed as means ± standard deviation. Statistical analysis was performed with Student’s t-test or one-way analysis of variance followed by Bonferroni post-hoc analysis where appropriate. *p* < 0.05 was considered statistically significant.

## Results

### SOX30 expression is lower in prostate cancer tissues and cell lines

**Fig. 1:**
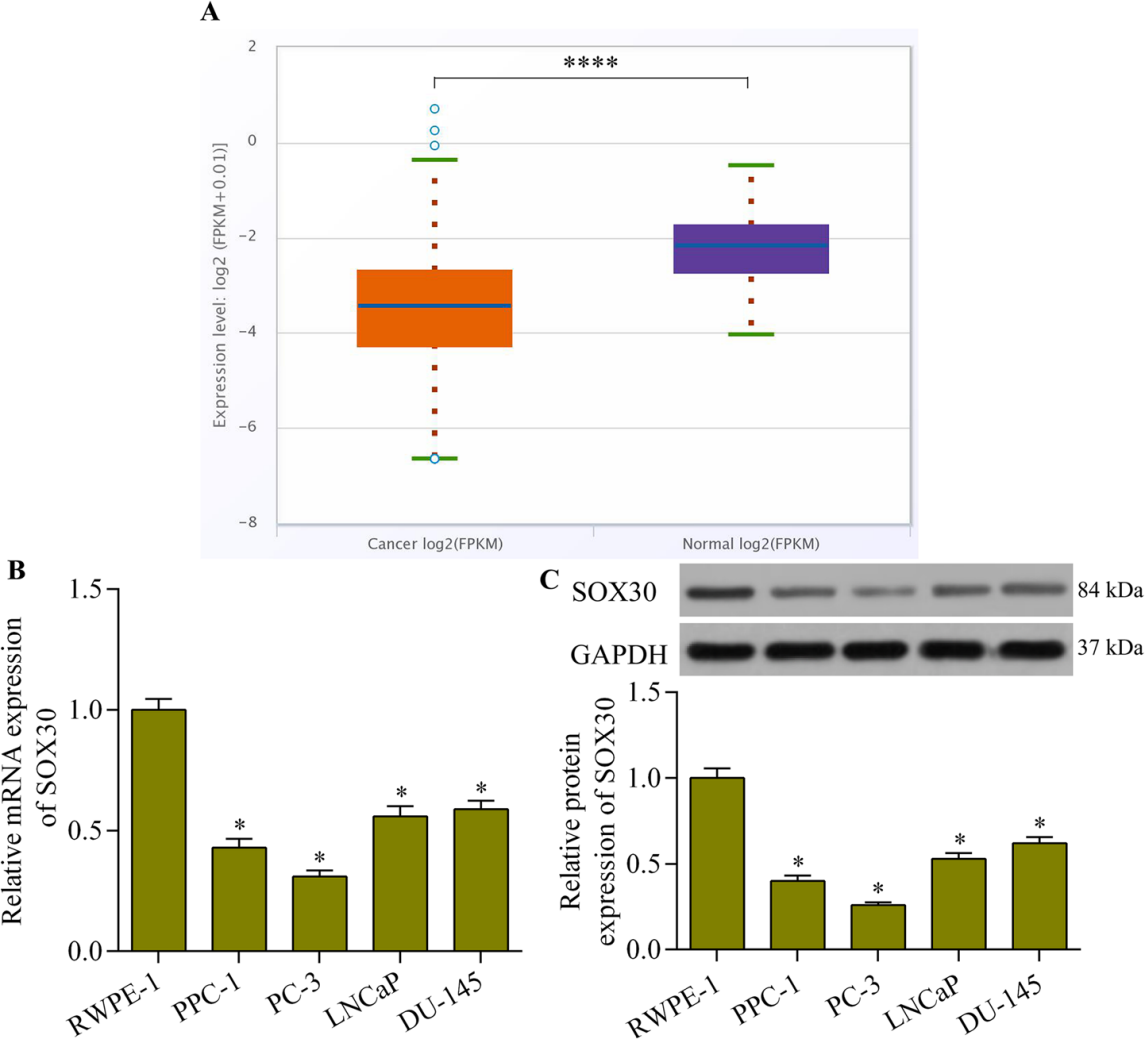
SOX30 expression was lower in prostate cancer cells than in normal prostate epithelial cells. The prostate cancer cell lines for the experiments were PPC-1, PC-3, LNCaP and DU-145. The normal prostate epithelial cell line RWPE-1 served as the control. **a** SOX30 expression in prostate cancer (*n* = 499) and normal (*n* = 52) samples was determined with the starBase Pan-Cancer Analysis Platform. ****p < 0.0001. **b** Relative SOX30 mRNA expression in prostate cancer cell lines was examined using quantitative real-time PCR (*n* = 5, *p < 0.05 versus RWPE-1). **c** SOX30 protein expression in cancer cell lines was detected using western blotting (*n* = 5, *p < 0.05 versus RWPE-1)

We analyzed SOX30 expression in prostate cancer tissues using the Pan-Cancer Analysis Platform of starBase v3.0 (http://starbase.sysu.edu.cn/) [27]. Interestingly, SOX30 is commonly downregulated in prostate cancer compared with its level in normal tissues (Fig. 1a). We also examined the SOX30 expression pattern in a panel of prostate cancer cell lines. Quantitative real-time PCR analysis showed that SOX30 mRNA expression was significantly lower in prostate cancer cell lines than in normal prostate epithelial cell lines (Fig. 1b). SOX30 protein expression was also consistently lower in prostate cancer cell lines compared with normal prostate epithelial cells (Fig. 1c). These results indicate that SOX30 levels decrease in prostate cancer.

### SOX30 overexpression restricts the progression and invasion of prostate cancer cells

**Fig. 2:**
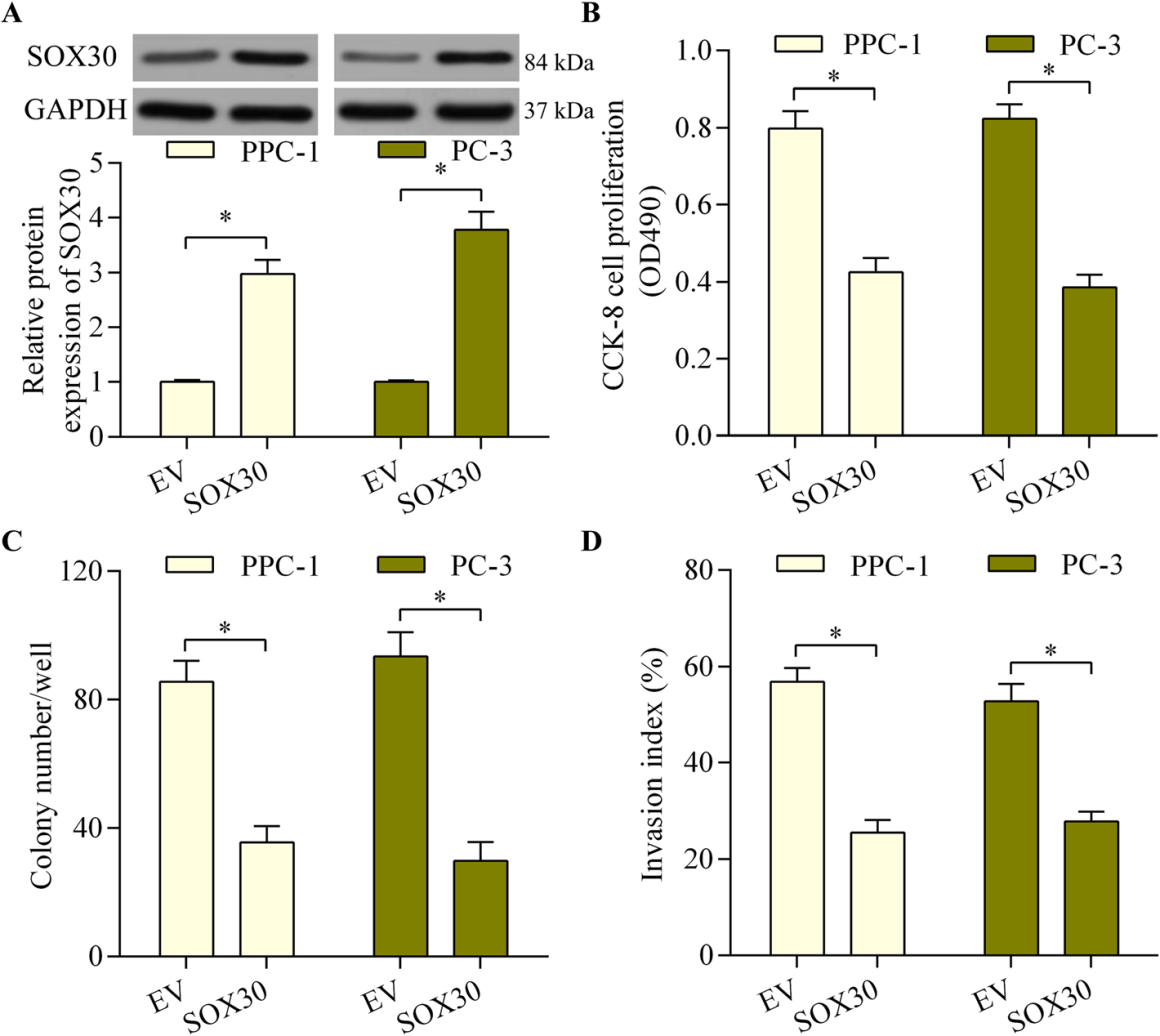
SOX30 overexpression repressed prostate cancer cell proliferation and invasion. **a** PPC-1 and PC-3 cells were transfected with pcDNA3.1/SOX30 vector or empty vector (EV) for 48 h and SOX30 protein expression was examined via western blotting. **b** The effect of SOX30 overexpression on prostate cancer cell proliferation was determined with the CCK-8 assay after transection of cells with the pcDNA3.1/SOX30 vector for 48 h. **c** The effect of SOX30 overexpression on prostate cancer cell colony-forming ability was assessed with a colony formation assay. PPC-1 and PC-3 cells were transfected with pcDNA3.1/SOX30 vector for 48 h and then cultured for 14 days to form colonies. **d** The effect of SOX30 overexpression on prostate cancer cell invasive potential was evaluated using a transwell Matrigel invasion assay. PPC-1 and PC-3 cells were transfected with pcDNA3.1/SOX30 vector for 48 h. The transwell Matrigel invasion assay ran for 24 h. (*n* = 5, *p < 0.05)

SOX30 gain-of-function experiments gave more insight into its biological function in prostate cancer. Its expression was significantly elevated after SOX30 expression vector transfection, as confirmed using western blotting (Fig. 2a). Its overexpression markedly reduced the proliferative ability of prostate cancer cells (Fig. 2b). Moreover, this overexpression significantly suppressed the colony-forming capability of prostate cancer cells (Fig. 2c). SOX30 overexpression also significantly decreased the invasive potential of prostate cancer cells (Fig. 2d). These results suggest a tumor-suppressive function for SOX30 in prostate cancer via inhibition of cell proliferation and invasion.

### SOX30 is a miR-653-5p target gene

**Fig. 3:**
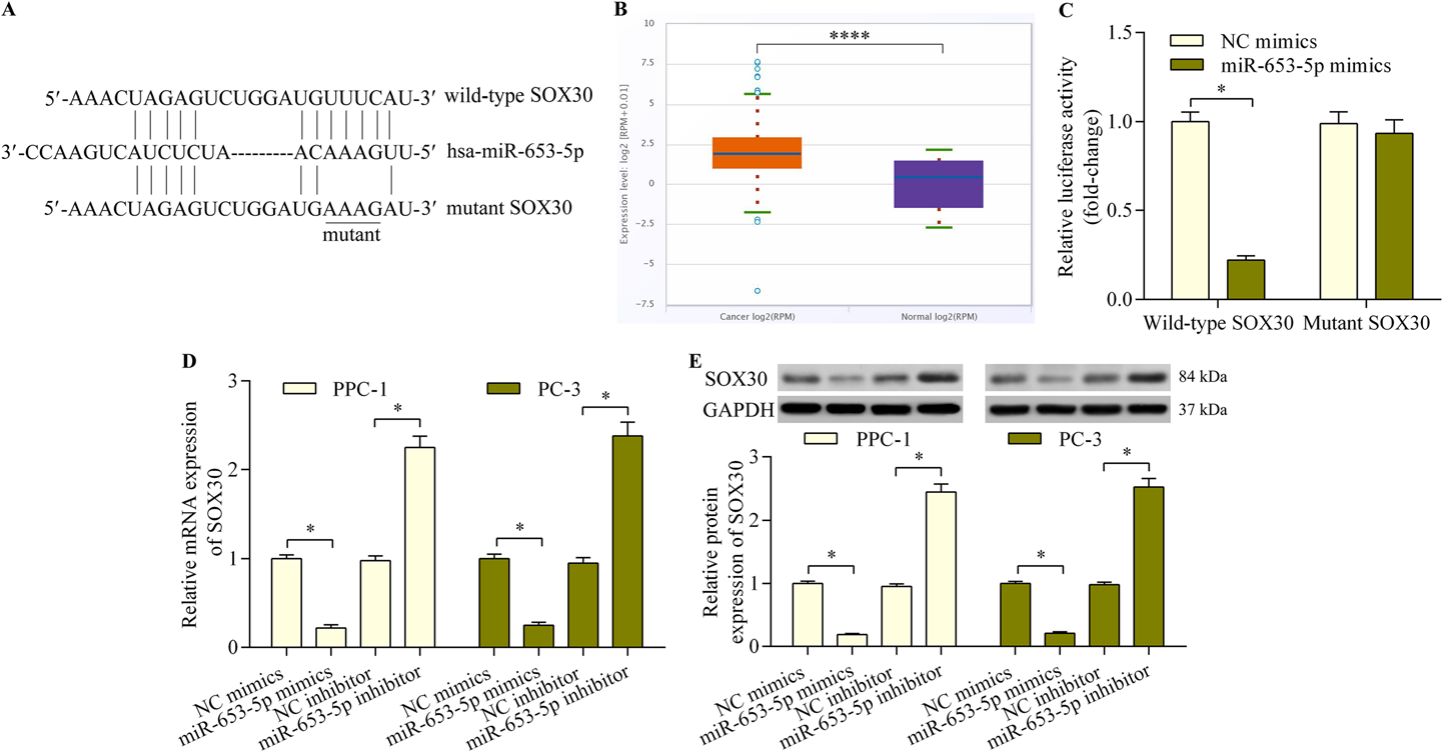
SOX30 is an miR-653-5p target gene. **a** Sequence alignment of the miR-653-5p-binding site within the SOX30 3′-UTR. **b** MiR-653-5p expression in prostate cancer (*n* = 495) and normal (*n* = 52) samples was determined with the starBase Pan-Cancer Analysis Platform. PPC-1, PC-3, LNCaP and DU-145 were the prostate cancer cell lines. The normal prostate epithelial cell line RWPE-1 served as the control. ****p < 0.0001. **c** The interaction between miR-653-5p and SOX30 3′-UTR was assessed with a dual-luciferase reporter assay using 293 T cells. SOX30 3′-UTR reporter plasmids and miR-653-5p mimics or NC mimics were co-transfected into 293 T cells and incubated for 48 h before determination of luciferase activity. *n* = 5, *p < 0.05. **d** and **e** PPC-1 and PC-3 cells were transfected with miR-653-5p mimics, inhibitor or NC mimics/inhibitor for 48 h. SOX30 mRNA (**d**) and protein (**e**) expressions were respectively determined using quantitative real-time PCR and western blot analysis (*n* = 5, *p < 0.05)

Since SOX30 expression decreased in prostate cancer, we explored the underlying mechanism responsible for this reduction. Through bioinformatics analysis, we found that SOX30 is a potential target gene of miR-653-5p, a tumor-associated miRNA [28, 29]. The SOX30 3′-UTR contains a putative miR-653-5p-binding site (Fig. 3a). Interestingly, miR-653-5p expression is significantly upregulated in prostate cancer tissues, as determined with Pan-Cancer Analysis Platform of starBase v3.0 (http://starbase.sysu.edu.cn/; Fig. 3b).

Next, we performed a dual-luciferase reporter assay to verify whether miR-653-5p directly binds to the SOX30 3′-UTR. miR-653-5p overexpression significantly reduced the luciferase activity of the wild-type SOX30 3′-UTR reporter, but had no obvious effect on the mutant SOX30 3′-UTR reporter (Fig. 3c). Moreover, transfection of the miR-653-5p mimics into prostate cancer cells significantly decreased SOX30 expression, while the miR-653-5p inhibitor markedly increased SOX30 expression (Fig. 3d and e). Collectively, these results indicate that SOX30 is a miR-653-5p target gene in prostate cancer.

### miR-653-5p inhibition repressed prostate cancer cell proliferation and invasion

**Fig. 4:**
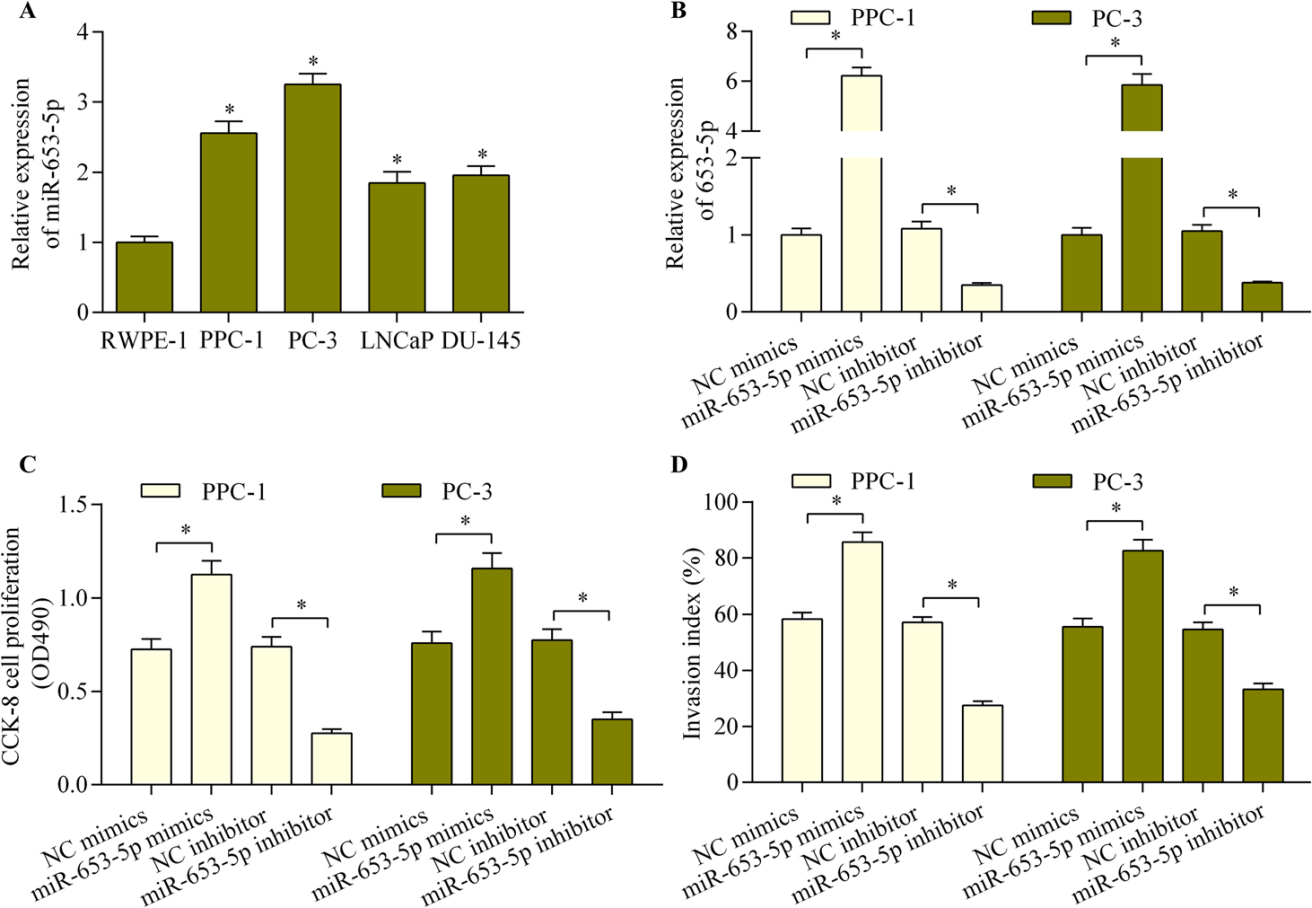
MiR-653-5p inhibition repressed prostate cancer cell proliferation and invasion. **a** The relative miR-653-5p expression in prostate cancer cell lines was examined using quantitative real-time PCR (*n* = 5, *p < 0.05 versus RWPE-1). **b** PPC-1 and PC-3 cells were transfected with miR-653-5p mimics, inhibitor or NC mimics/inhibitor for 48 h. The relative miR-653-5p expression was determined using quantitative real-time PCR. **c** Cell proliferation was measured with the CCK-8 assay after transfection with miR-653-5p mimics, inhibitor or NC mimics/inhibitor for 48 h. **d** PPC-1 and PC-3 cells were transfected with miR-653-5p mimics, inhibitor, or NC mimics/inhibitor for 48 h. The transwell Matrigel invasion assay to determine cell invasion ran for 24 h (*n* = 5, *p < 0.05)

To investigate whether miR-653-5p is involved in prostate cancer, we determined the regulatory effect of miR-653-5p on prostate cancer cell proliferation and invasion. MiR-653-5p expression was significantly upregulated in prostate cancer cell lines (Fig. 4a). As expected, miR-653-5p overexpression promoted the proliferation and invasion of prostate cancer cells, while its inhibition significantly restricted these measures (Fig. 4b through d). These results suggest that miR-653-5p inhibition represses prostate cancer cell proliferation and invasion.

### SOX30 reduced the activation of Wnt/β-catenin signaling in prostate cancer

**Fig. 5:**
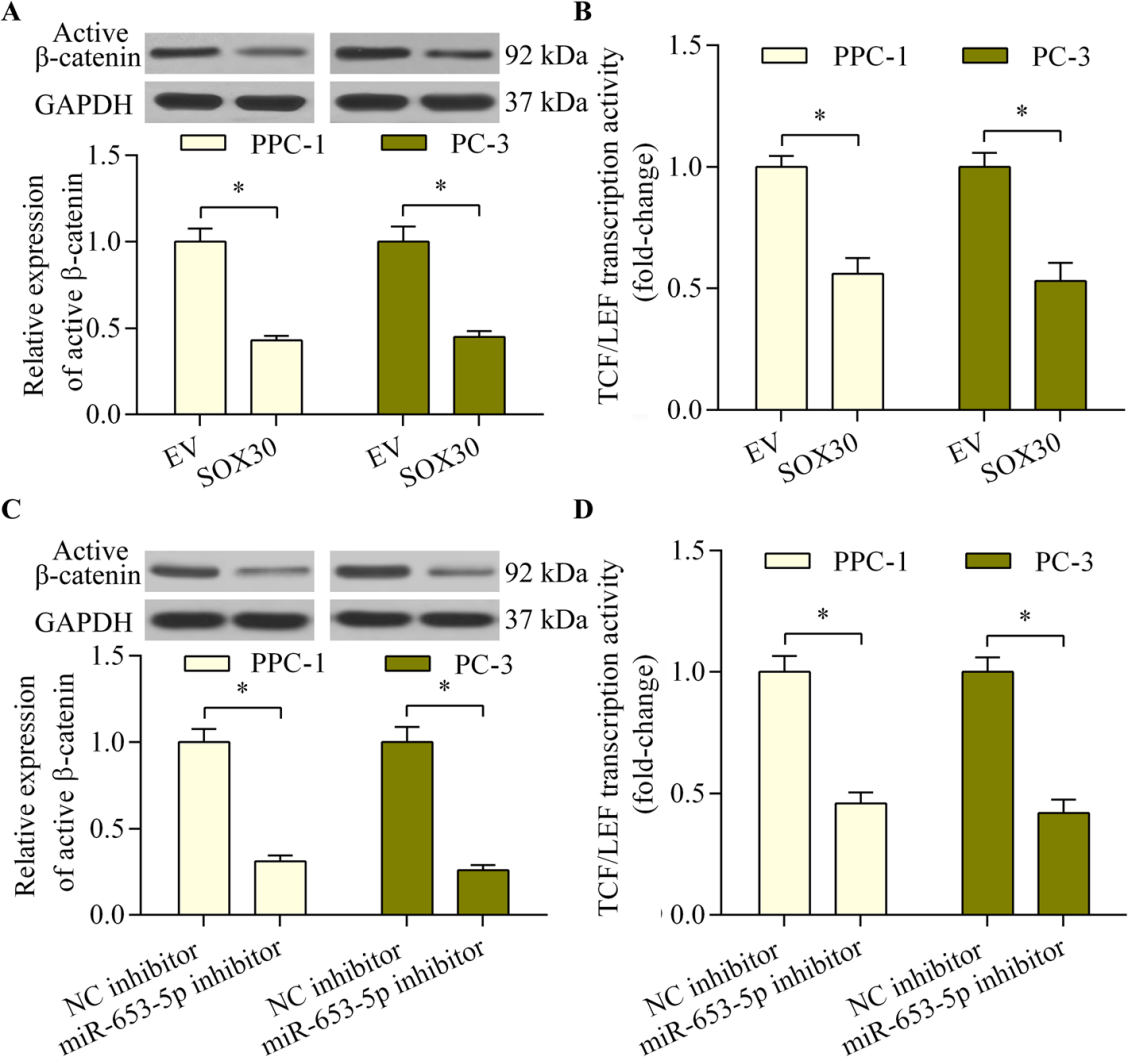
SOX30 inhibited the activation of Wnt/β-catenin signaling in prostate cancer cells. **a** The effect of SOX30 overexpression on active β-catenin expression was determined using western blotting. PPC-1 and PC-3 cells were transfected with SOX30 expression vector and incubated for 48 h. **b** The effect of SOX30 overexpression on Wnt/β-catenin signaling was assessed with a TCF/LEF luciferase reporter assay. PPC-1 and PC-3 cells were co-transfected with SOX30 expression vector and TCF/LEF luciferase reporter vector and incubated for 48 h. **c** The effect of miR-653-5p inhibition on active β-catenin expression was measured using western blotting. PPC-1 and PC-3 cells were transfected with miR-653-5p inhibitor or NC inhibitor and incubated for 48 h. **d** The effect of miR-653-5p inhibition on Wnt/β-catenin signaling was assessed with a TCF/LEF luciferase reporter assay. PPC-1 and PC-3 cells were co-transfected with TCF/LEF luciferase reporter vector and miR-653-5p inhibitor or NC inhibitor and incubated for 48 h (*n* = 5, *p < 0.05)

We next investigated the molecular basis for SOX30 regulation of prostate cancer cell proliferation and invasion. Previous studies reported that SOX30 has a significant impact on Wnt/β-catenin signaling [18, 19], which plays a crucial role in prostate cancer progression [30]. Therefore, we evaluated the regulatory effect of SOX30 on Wnt/β-catenin signaling in prostate cancer cells. SOX30 overexpression significantly downregulated active β-catenin expression and reduced the transcription activity of TCF/LEF (Fig. 5a and b). Moreover, miR-653-5p inhibition also significantly decreased Wnt/β-catenin signaling activation (Fig. 5c and d). These results indicate that SOX30 overexpression or miR-653-5p inhibition suppresses the activation of Wnt/β-catenin signaling in prostate cancer cells.

### SOX30 silencing reversed the miR-653-3p inhibition-mediated anti-tumor effect in prostate cancer cells

**Fig. 6:**
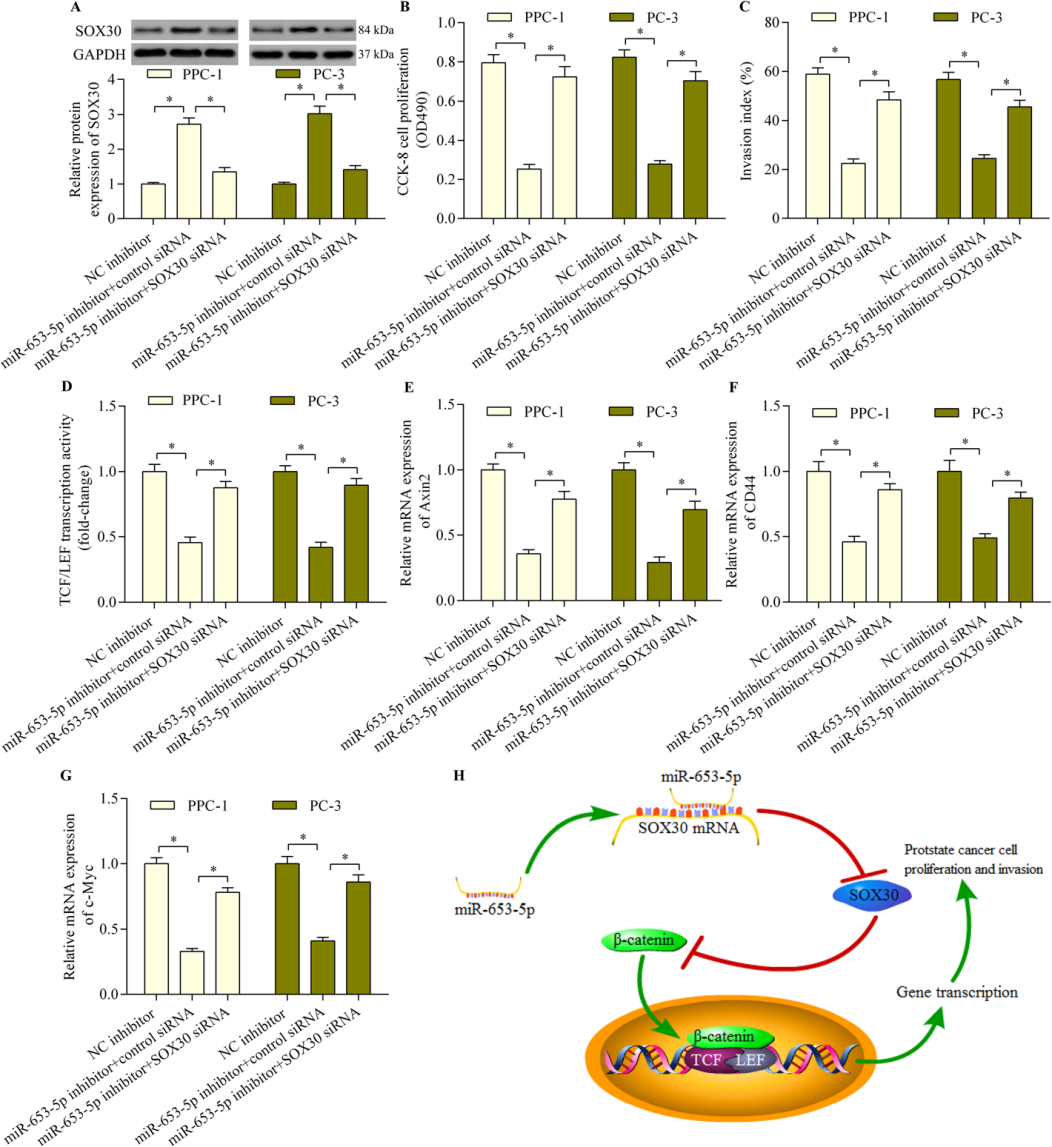
SOX30 silencing reversed the miR-653-3p inhibition-mediated anti-tumor effect in prostate cancer cells. **a** PPC-1 and PC-3 cells were co-transfected with miR-653-5p inhibitor and SOX30 siRNA for 48 h. SOX30 protein expression was determined using western blotting. **b** Cell proliferation was examined with the CCK-8 assay after co-transfection with miR-653-5p inhibitor and SOX30 siRNA for 48 h. **c** PPC-1 and PC-3 cells were co-transfected with miR-653-5p inhibitor and SOX30 siRNA for 48 h. A transwell Matrigel invasion assay was run for 24 h to determine cell invasion. **d** Wnt/β-catenin signaling was assessed with a TCF/LEF luciferase reporter assay. PPC-1 and PC-3 cells were co-transfected with TCF/LEF luciferase reporter vector, miR-653-5p inhibitor and SOX30 siRNA and incubated for 48 h before determination of luciferase activity. **e**, **f** and **g** PPC-1 and PC-3 cells were co-transfected with miR-653-5p inhibitor and SOX30 siRNA for 48 h, and relative mRNA expression levels of Axin2 (**e**), CD44 (**f**), and c-Myc (**g**) were determined using quantitative real-time PCR (*n* = 5, *p < 0.05). **h** A graphical model of the miR-653-5p–SOX30–Wnt/β-catenin axis in regulating prostate cancer cell proliferation and invasion

To validate whether SOX30 acts as a functional miR-653-5p target in regulating prostate cancer cell proliferation, invasion and Wnt/β-catenin signaling, we determined the effect of SOX30 silencing on the miR-652-3p inhibition-mediated anti-tumor effect. SOX30 siRNA transfection significantly abrogated the promotional effect of miR-653-5p inhibition on SOX30 expression (Fig. 6a). As expected, SOX30 silencing significantly reversed the inhibitory effect of miR-653-5p inhibition on prostate cancer cell proliferation and invasion (Fig. 6b and c). Moreover, SOX30 silencing markedly abrogated the suppressive effect of miR-653-5p inhibition on Wnt/β-catenin signaling activation (Fig. 6d) and the expression of Wnt/β-catenin target genes, including Axin2, CD44 and c-Myc (Fig. 6e through g). Overall, these results suggest that miR-653-5p inhibition exerts an anti-tumor effect in prostate cancer cells by upregulating SOX30.

## Discussion

This paper is the first to report a tumor suppressive function for SOX30 in prostate cancer. We found that SOX30 expression was lower in prostate cancer cells than in normal tissues and that it is a miR-653-5p target gene. SOX30 overexpression or miR-653-5p inhibition markedly repressed the proliferation and invasion of prostate cancer cells through downregulation of Wnt/β-catenin signaling. Our findings highlight the involvement of the miR-653-5p–SOX30–Wnt/β-catenin signaling axis in prostate cancer progression (Fig. 6h).

SOX30 is essential for male mammalian spermatogonial differentiation, spermatogenesis and testis development [31–34]. Its dysregulation is associated with tumorigenesis in multiple tumors [10]. It is highly expressed in normal and peri-tumoral lung tissues, but it is reduced in primary lung tumor tissues and lung cancer cell lines [12]. Increased SOX30 expression correlates with long survival time and suggests favorable survival outcomes for lung cancer patients [13]. Its overexpression restricts the growth and metastasis of lung cancer in vitro and in vivo [18, 35]. Notably, decreased SOX30 expression occurs in hepatocellular carcinoma, acute myeloid leukemia, malignant lymphomas, ovarian cancer and bladder cancer [14–17, 36]. These findings indicate a tumor-suppressive function for SOX30.

In this study, we found decreased SOX30 expression in prostate cancer tissues and cell lines and showed that its overexpression significantly represses the proliferation and invasion of prostate cancer cells. These data support a tumor-suppressive function for SOX30 in prostate cancer. Therefore, SOX30 may serve as a potential anticancer target for prostate cancer treatment.

Although the tumor-suppressive function of SOX30 is well characterized, some key questions remain unanswered regarding the decreased SOX30 expression in tumor tissues. Intriguingly, recent studies revealed that decreased SOX30 expression is associated with miRNA dysregulation in tumor tissues. MiR-125b targets the SOX30 3′-UTR, and high miR-125b expression correlates with low SOX30 expression in the malignant lymphomas [36]. Moreover, SOX30 is an miR-645 target gene in hepatocellular carcinoma and colon cancer [14, 37]. However, whether SOX30 expression is regulated by miRNAs in prostate cancer remains unknown.

Our study identified SOX30 as an miR-653-5p target gene in prostate cancer. Moreover, our data show that knockdown of SOX30 partially reverses the miR-653-5p downregulation-induced tumor-suppressive effect in prostate cancer cells, indicating that SOX30 is a functional target of miR-653-5p in prostate cancer. However, our results cannot rule out the possibility that SOX30 may be regulated by other miRNAs that target it, such as miR-125b and miR-645. Further investigation is needed to elaborate on the regulation of SOX30 by miRNAs in prostate cancer. Nevertheless, our study suggests that the miR-653-5p–SOX30 axis may be involved in the progression of prostate cancer.

The function of miR-653-5p is underreported. It regulates the proliferation and apoptosis of thymocytes in myasthenia gravis [38]. It has more recently been identified as a cancer-related miRNA for several tumors. MiR-653-5p upregulation inhibits breast cancer cell growth and promotes apoptosis by downregulating zinc-finger E-box-binding homeobox 2 [39]. In neuroblastoma, miR-653-5p targets STAT2 to regulate neuroblastoma cell proliferation and invasion [29]. It functions as a tumor-suppressive miRNA in lung cancer by targeting TIAM1 to inhibit cell proliferation and invasion [29]. These findings indicate that miR-653-5p may exert a tumor-suppressive function in these tumor types.

However, our study revealed an oncogenic role for miR-653-5p in prostate cancer. It is significantly upregulated in prostate cancer tissues and cell lines, and its inhibition markedly reduces cell proliferation and invasion. We also identified that the miR-653-5p oncogenic effect in prostate cancer is associated with its inhibitory effect on SOX30. The expression and function of miRNAs can be critically dependent on the tissue and cell type involved, but our study suggests that miR-653-5p targets SOX30 and thus participates in prostate cancer progression. Targeting miR-653-5p to modulate SOX30 expression may represent a novel therapeutic strategy for prostate cancer.

Interestingly, SOX30 exerts its anti-tumor effect through Wnt/β-catenin signaling inactivation [40]. SOX30 can inhibit β-catenin expression [19, 35], directly interact with β-catenin, and abrogate β-catenin binding to TCF. These actions inhibit Wnt/β-catenin signaling [18]. In line with these findings, our study showed that SOX30 overexpression contributes to reduced Wnt/β-catenin signaling in prostate cancer cells. Considering that Wnt/β-catenin signaling is highly activated and contributes to prostate cancer progression [30], targeting SOX30 to inhibit Wnt/β-catenin signaling may have a potential application in prostate cancer therapy.

## Conclusions

The findings of our study demonstrate that SOX30 is a tumor-suppressive gene in prostate cancer and an miR-653-5p target. Our results suggest that inhibiting miR-653-5p restricts the proliferation and invasion of prostate cancer cells due to the inhibition of Wnt/β-catenin signaling through the miRNA’s targeting of SOX30. Our data highlight the involvement of the miR-653-5p–SOX30–Wnt/β-catenin signaling axis in the progression of prostate cancer and provide novel insights into the molecular pathogenesis of prostate cancer. MiR-653-5p and SOX30 may be novel and promising targets for prostate cancer.

## Data Availability

The data in this study are available from the author for correspondence upon reasonable request.

## Abbreviations

3′-UTR: 3′-untranslated region
CCK-8: Cell counting kit − 8
FBS: Fetal bovine serum
miR-653-5p: MicroRNA-653-5p
miRNAs: MicroRNAs
SOX30: Sex determining region Y-box containing gene 30
